# Supplementary material for: Association between the novel inflammatory marker white blood cell count-to-mean platelet volume ratio and metabolic syndrome: A cross-sectional study based on NHANES 2011–2020
Source: PLoS One. 2026 Apr 2;21(4):e0344017. doi: 10.1371/journal.pone.0344017 (PMC13046166; doi:10.1371/journal.pone.0344017)
Supplement: Attachment — Submitted filename: Response to Reviewers.docx [file pone.0344017.s002.docx]

Response to Reviewers

Dear Editor,

Thank you for taking the time to review my manuscript amidst your busy schedule. I have carefully addressed the comments you provided and made the corresponding revisions. The detailed revisions are as follows:

1. Please ensure that your manuscript meets PLOS ONE's style requirements, including those for file naming.

In the revised manuscript, we have modified the article according to PLOS ONE's style requirements.

2. Thank you for stating the following in the Competing Interests section: NO authors have competing interests

We have included this explanation in the revised manuscript.

3. Your ethics statement should only appear in the Methods section of your manuscript. If your ethics statement is written in any section besides the Methods, please move it to the Methods section and delete it from any other section. Please ensure that your ethics statement is included in your manuscript, as the ethics statement entered into the online submission form will not be published alongside your manuscript.

In accordance with the requirements, we have relocated the ethics statement to the Methods section in the revised manuscript.

4. Thank you for uploading your study's underlying data set. Unfortunately, the repository you have noted in your Data Availability statement does not qualify as an acceptable data repository according to PLOS's standards.

At this time, please upload the minimal data set necessary to replicate your study's findings to a stable, public repository (such as figshare or Dryad) and provide us with the relevant URLs, DOIs, or accession numbers that may be used to access these data. For a list of recommended repositories and additional information on PLOS standards for data deposition, please see https://journals.plos.org/plosone/s/recommended-repositories.

We have uploaded the data to Figshare in accordance with the requirements. Kindly verify whether it meets the journal’s standards.

URL：https://figshare.com/account/articles/29557250?file=56236955

DOI ：10.6084/m9.figshare.29557250

5. Please upload a new copy of Figure 2 as the detail is not clear. Please follow the link for more information: https://blogs.plos.org/plos/2019/06/looking-good-tips-for-creating-your-plos-figures-graphics/"

We have re-uploaded Figure 2.

6. Please review your reference list to ensure that it is complete and correct. If you have cited papers that have been retracted, please include the rationale for doing so in the manuscript text, or remove these references and replace them with relevant current references. Any changes to the reference list should be mentioned in the rebuttal letter that accompanies your revised manuscript. If you need to cite a retracted article, indicate the article’s retracted status in the References list and also include a citation and full reference for the retraction notice.

We have thoroughly reviewed the reference list and confirmed that it contains no retracted publications.

Dear Reviewers,

I sincerely appreciate your thorough and thoughtful review of my manuscript and the valuable suggestions you have provided. Your insightful feedback has been instrumental in enhancing the quality of my manuscript. I have now revised the manuscript in accordance with your recommendations. The detailed revisions are as follows:

1. • Title: The title does not clearly indicate that the study is cross-sectional.

• Abstract: The abstract provides a concise summary of the study, including the background, objectives, methods, key findings, and conclusions. However, it would benefit from a clearer mention of the study design (cross-sectional). Besides, I suggest revising the conclusion to better emphasize on performing of prospective studies in the conclusion section.

•In the revised manuscript, we modified the title to clearly indicate that this is a cross-sectional study.

•In the revised abstract, we added in the Methods section that this is a cross-sectional study. Furthermore, in the conclusion section, we emphasized the need for conducting prospective studies.

2. Introduction：First paragraph: The same references (first) should not be used for consecutive sentences; the first reference should be removed.

In the revised manuscript, we have removed the first reference.

3. Methodology

• The study design is appropriate for the research objectives. However, the exclusion criteria regarding WBC could be more clearly explained. Specifically, I recommend elaborating on the exclusion criteria of the critical influencing factors, including chronic inflammatory disease, cancer, drugs, and others that could affect WBC measurement accuracy.

• Number of participants is different from flowchart and abstract.

• The definition of cigarette smoking could be clarified.

•In the revised manuscript, we added relevant explanations in the Data Collection section of the Methods.

•We appreciate your careful observation, and have corrected the number of participants in the Methods section to align with the flowchart and abstract.

•In the revised manuscript, the Methods section includes an added description of smoking classification.
